# Editorial: Recent progress in polymer-based biomaterials as adhesives

**DOI:** 10.3389/fbioe.2023.1305531

**Published:** 2023-12-12

**Authors:** Xiaoyuan Li, Jianxun Ding, Denghui Xie, Shasha He, Jinshan Guo

**Affiliations:** ^1^ Northeast Normal University, Changchun, China; ^2^ School of Chemistry, Northeast Normal University, Changchun, China; ^3^ Changchun Institute of Applied Chemistry, Chinese Academy of Sciences (CAS), Changchun, China; ^4^ Third Affiliated Hospital, Southern Medical University, Guangzhou, China; ^5^ Xiamen University, Xiamen, China; ^6^ Southern Medical University, Guangzhou, China

**Keywords:** adhesive, biomaterials, tissue engineering, wound healing, biological applications

## Introduction

The high incidence rate of refractory wounds derived from chronic diseases and traumatic injuries still threatens the life of human beings, especially when the unclosed wounds are of large areas and accompanied by microbial infection. Adhesive biomaterials are widely applied as wound dressings due to their easy operation and significant potential to reduce skin scarring, usually caused by the closure of surgical wounds with sutures or staples. Although fibrin glue or some poly(ethylene glycol) (PEG)-based adhesives have been utilized in clinical applications and proved effective, source safety and weak interaction trigger scientists to investigate more non-animal origin adhesives with suitable adhesion and cohesion force (Jain and Wairkar, 2019).

It is worth noting that research on these bioinspired and biocompatible adhesives is no longer limited to traditional wound repair, and it has extended to various tissue engineering fields, such as ophthalmology, dentistry, orthopedics, and bone regeneration (Li et al., 2023). Continuous efforts are now devoted to improving interfacial interaction between different tissues and wet adhesion capacity (Li et al., 2020). Predictably, the biomaterials-based adhesives either adhere to tissues firmly or be peeled off easily on demand, which would significantly elevate the regeneration process of patients. Herein, Zheng et al. summarized the characteristics that an ideal retinal patch must have to create a stable and safe seal around the retinal breaks for the repair of rhegmatogenous retinal detachment, including good biocompatibility, strong and stable persistent adhesion, flexibility, easy delivery, and appropriate biodegradability. Zhao et al. utilized urushiol from lacquer sap as an additive to surface acid etchant on dentin. Treatments with urushiol could improve dentin biostability and durability by interacting with collagen and inactivating matrix metalloproteinase activity. The application of the etchants containing urushiol also exhibited instinctive antibacterial property against the common cariogenic bacteria *Streptococcus mutans*, *Streptococcus sanguinis*, and *Streptococcus gordonii*, as well as the biofilm in the complicated oral microecology.

As one important application of adhesives, biological adhesives for bone fracture fixation and defect repair may probably replace traditional invasive materials, such as steel plates or screws, and revolutionize orthopedic surgeries (Xu et al., 2020). In order to mimic the composite constitution of natural bone, most scaffolds for bone regeneration are designed to be composed of organic and inorganic materials, in combination with cells or bioactive components. Polymers whether melt forming or hydrogel molding, usually be present in the bulk mixture or surface modification, and they serve as bioadhesives and supporting scaffold to adhere the inorganic components together and load functional ingredients. Therefore, biomaterials for bone regeneration related disease therapy were also included in this Research Topic. Liu et al. summarized the combination of stem cells or extracellular vesicles based on stem cells with biomaterials for the treatment of intervertebral disc degeneration (IDD). Orthopedic implant-related infection leads to formation of dead bone and cavities, hinders the regeneration process and even causes high rate of amputation and mortality. Emergence of bacterial resistance and biofilm formation have made people aware of the overuse and side effects of systemic antibiotics. Shuaishuai et al. evaluated unique antimicrobial materials including organic materials, inorganic materials, and composite materials and their performance on orthopedic-related infections.

## Perspectives

The goal of this Research Topic is designed to shed light on the advanced adhesives and their multiple applications in clinical fields. Adhesives are no longer restricted to blood clotting, wound dressing and sealing, but appear as a necessary biomaterial component for extensive applications. In addition to tissue regeneration endowed by bio-scaffolds, antibacterial and immune regulation are of vital importance as well. This article collection will probably provide guidance to researchers and inspire the innovative investigation in biomedical field.
